# Review of the Results of WT1 Peptide Vaccination Strategies for Myelodysplastic Syndromes and Acute Myeloid Leukemia from Nine Different Studies

**DOI:** 10.3389/fimmu.2015.00036

**Published:** 2015-02-04

**Authors:** Antonio Di Stasi, Antonio M. Jimenez, Kentaro Minagawa, Mustafa Al-Obaidi, Katayoun Rezvani

**Affiliations:** ^1^Stem Cell Transplantation and Cell Therapy Unit, The University of Alabama at Birmingham, Birmingham, AL, USA; ^2^Stem Cell Transplantation and Cell Therapy Unit, Rush University Medical Center, Chicago, IL, USA; ^3^Stem Cell Transplantation and Cell Therapy Unit, The University of Texas MD Anderson Cancer Center, Houston, TX, USA

**Keywords:** WT1, peptide vaccine, MDS/AML, TAA, active immunotherapy

## Abstract

We performed a systematic review of data from nine clinical trials of WT1 peptide vaccination in patients with myelodysplastic syndromes and/or acute myeloid leukemia (MDS/AML), published between 2004 and 2012. A total of 51 patients were eligible for analysis. Vaccination with WT1 peptides proved safe and feasible in patients with MDS/AML, in studies from different institutions. Additionally, clinical responses and clinical benefit were observed, with some patients achieving and maintaining remission long-term (more than 8 years). A significant correlation between induction of WT1-specific T cells and normalization/reduction of WT1 mRNA levels and progression-free survival was noted in a number of studies. However, larger studies are warranted to confirm these results. Interestingly, the majority of trials reported the presence of WT1-specific T cells with limited or absent functionality prior to vaccination, which increased in frequency and function after vaccination. In conclusion, WT1 peptide vaccination strategies were safe in this heterogeneous group of patient with MDS/AML. Larger and more homogeneous studies or randomized clinical trials are needed to quantify the contribution of WT1 peptide vaccines to clinical responses and long-term survival.

## Introduction

Allogeneic hematopoietic stem cell transplantation (HSCT) remains the unique curative option for patients with myelodysplastic syndromes (MDS) and/or acute myeloid leukemia (AML) at high risk of relapse (1).

However, considering HSCT-related morbidity and mortality risks it is a suitable therapeutic option only for younger patients (generally up to 70 years of age) without significant comorbidities (2, 3). Hypomethylating agents are now the first-line treatment for patients with higher-risk MDS not eligible for HSCT and are being used for older patients with AML (4), however, since the prognosis of patients who lose response or progress while on hypomethylating agents is extremely poor (2) alternative strategies are needed. One approach can consist in boosting immunity toward tumor associated antigens (TAAs) by the mean of peptide vaccination. Vaccination would allow the induction of humoral and cellular adaptive immune responses to specific antigens, and an optimal cancer vaccine should prompt the activation of antigen-specific CD3^+^CD4^+^ and CD3^+^CD8^+^ T-lymphocytes.

An ideal leukemia TAA to be employed in anti-cancer vaccination strategies should be expressed on leukemic progenitors, be intrinsic to leukemic survival so that tumor escape by down-regulation of the antigen cannot occur, and induce a strong cytotoxic response. Over-expressed/aberrantly expressed cellular proteins, such as proteinase-3 PR1 peptide (PR1) (5), Wilms’ Tumor-1 (WT1) (5–14), or receptor for hyaluronan-mediated motility (RHAMM) (15), and the altered cell surface glycoprotein Mucin-1 (MUC1) (5) have been evaluated in phase I/II clinical trials of active immunotherapy, either alone or in combination as possible target antigens, with promising results.

The *WT1* gene located on chromosome 11p13 (16) encodes a zinc finger transcription factor that plays an important role in cell growth and differentiation (17). Expression of the WT1 protein is restricted to a limited set of tissues, including the gonads, uterus, kidney, and mesothelium, and to progenitor cells in various types of tissues (18). WT1 knock-out mice were found to have defects in the urogenital system and died on ED 13.5, probably due to heart failure (19). The *WT1* gene is highly expressed by the majority of AML and acute lymphoid leukemia (20). Furthermore, in chronic myeloid leukemia (21) and MDS (22), *WT1* mRNA expression levels were shown to increase with disease progression. Although originally defined as a tumor suppressor gene (23, 24), accumulating evidence suggests that WT1 has an oncogenic role in leukemogenesis and tumorigenesis (25), as inhibition of *WT1* gene expression resulted in suppression of leukemia growth *in vitro*, whereas its forced expression resulted in leukemia induction in mice (26, 27). Perhaps most relevant to the clinical setting, immunization of mice with WT1 peptides was shown to induce anti-tumor activity without inhibiting engraftment of normal CD34^+^ hematopoietic progenitor cells (28). The selectivity of WT1-specific human T cells as effectors against WT1 expressing targets has also been shown *in vitro* (29) and several epitopes, including helper T cell epitopes, have entered clinical trials (30).

Most TAAs are aberrantly expressed self-proteins, and T cells directed against these antigens typically express low-affinity T cell receptors as a consequence of the negative selection in the thymus. In contrast, when stimulated with low doses of foreign antigens in combination with noxious substances (adjuvants), the immune system is activated, leading to the generation of effector and memory T cells (31).

The success of a particular peptide vaccine to elicit an immune response is influenced by many parameters, including the presence of helper T cell epitopes, processing and presentation by professional antigen presenting cells (APCs), bio-distribution, influence of adjuvants, peptide length, peptide affinity, and mode of administration (Table 1).

**Table 1:** **Strategies to improve the efficacy of anti-tumor vaccination**.

Improve co-stimulation	1) Presence of appropriate cytokines
	2) Use of T-helper epitopes or DC agonists (TNF, TLR, and PADRE)
	3) Slow release vaccines
	4) Draining to local activated lymph nodes
	5) Avoid continuous or repeated administration, which can induce T-regulatory cells
	6) Peptide elongation
Prevent systemic spread	1) Attachment of lipid tails to peptides
	2) Linking APC activating compounds and antigen
	3) Adoption of linkers between cytotoxic and helper sequences
Reduce toxicity	1) Avoid quick and widespread bio-distribution (cytokine storm)
	2) Avoid high doses or repeated administration
	3) Identify bio-markers to predict and monitor toxicity

*TNF, tumor necrosis factor; TLR, toll like receptor; PADRE, T cell Pan DR epitope; APC, antigen presenting cells*.

This review, we will summarize the immunologic and clinical results of WT1 peptide vaccination approaches in patients with myelodysplastic syndromes and/or acute myeloid leukemia (MDS/AML) (31).

## Clinical Studies of WT1 Peptide Vaccines in MDS/AML

A detailed report of the clinical studies reviewed in this article is presented in Table 2 (5–14).

**Table 2:** **Summary of reviewed clinical trials**.

Diagnosis (*N*)	Disease status/[previous tx]	Epitope	Vax# (*range*); [adjuvant]	(*N*) Toxicity grade III-IV	Anti WT1 responses	Clinical responses		[Follow-up]/[response duration]	Reference
AML (1)	1PR [chemo]	WT1_126–134_	15; [KLH]	None	Yes	Yes	(1) Morphological/molecular CR	[46 weeks]/[30 weeks]	(6)
AML (17) MDS (2)	13 PD	WT1_126–134_	11(*4–27*); [KLH]	None	N/A	Yes	(1) CR, (13) SD, (4) PD, (1) Major neutrophil response	[NA]/[CR: 16 months; PFS SD 155D (101–571)]	(7)
	6 PR	
	2 EB [chemo]	
AML (12) MDS (2) other (12)	8 CR	Natural WT1_235–243_ vs. modified	3; [mISA51]	None	Yes	Yes	(5) Molecular CR (2) PR, (1) SD, (2) PD, (4) NE	[NA]	(8)
	4MRD	
	2 EB [NA]	
AML (3)	3 MRD [chemo]	Natural WT1_235–243_ vs. modified	Several; [mISA51]	None	Yes	Yes	(3) CR for >8 years	[90 months (90–94)]/[NA]	(9)
AML (5) MDS (2) other (1)	4CR [chemo]	PR1_169–177_ and WT1_126–134_	6; [mISA51]	None	Yes	Yes	(3) CCR, (2) SD, (2) relapse	[NA]/[SD: 180D (105–523)]	(11)
	1 RA	
	1 RARS [EPO/GCSF]	
AML (6) MDS (2)	6 CR [5 chemo, 1 allo-HSCT]	PR1_169–177_ and WT1_126–134_	6; [mISA51]	None	N/A	Yes	(2) CCR, (1) SD, (1) PD, (4) relapse	[NA]/[SD: 832D., CCR: 683D (587–779), TTR: 112D. (14–352)]	(10)
	1 RA	
	1 RARS [EPO/GCSF]	
AML (1) MDS (1)	1 AD [chemo]	WT1_235–243_	20; [mISA51]	None	N/A	Yes	(1) Morphological CR, (1) molecular CR	[NA]/[CR > 3 years]	(12)
	1 MRD [NA]	
AML (9)	9 MRD [chemo]	WT1_126–134_^A1#^ and WT1_427–445/331–352/122–140_^A1#^	9 (*6–12*); [mISA51]	None	Yes	Yes	(5) CCR, (4) relapse	[NA]/[DFS 31 months (10–121), mPFS not reached]	(13)
AML (4) other (5)	3 AD [NA]	WT1_126–134_ and PR3_169–177_ with PADRE/MUC1helper epitope	6; [CPG7909/mISA51]	(4) Erythema, (1) dyspnea, (2) fever	None	Yes	(2) SD, (2) PD	[84D]/[NA]	(5)

*N, number; tx, treatment; vax, vaccine; WT1, Wilms’ tumor-1; AML, acute myeloid leukemia; PR, partial response; KLH, keyhole limpet hemocyanin; chemo, chemotherapy; (C)CR; (continuous) complete remission; MDS, myelodysplastic syndromes; PD, progressive disease; EB, excess blasts; NA, not available; (D)PFS, (disease) progression-free survival; SD, stable disease; MRD, molecular residual disease; mISA51, montanide ISA51; RA(RS), refractory anemia (ringed sideroblasts); EPO, erythropoietin; GSCF, granulocyte colony stimulating factor; allo-HSCT, allogeneic hematopoietic stem cell transplantation; TTR, time to relapse; AD, active disease; A1^#^, mutated amino acid R126Y; PR3, proteinase-3; PADRE; T cell Pan DR epitope; MUC1, Mucin-1; CPG 7909, immunostimulatory toll like receptor 9 (TLR9) agonist oligodeoxynucleotide*.

In order to analyze survival outcomes after vaccination, we combined the results from seven reports published between 2004 and 2012 (5, 7, 9, 10, 12–14). Unduplicated observations were available for 55 out of 67 patients with MDS/AML: 4 patients were not evaluable for response, and therefore the final number of evaluable patients in our analysis was 51. A summary of the patients and their responses is detailed in Table 2. The majority of treated patients received also granulocyte monocyte colony stimulating factor injections, and the majority were vaccinated against an epitope recognized in the context of human leukocyte antigen HLA-A02-01; however, some studies employed HLA-A24-02 (36–39), and in one study peptide recognized in the context of HLA-A02-01 were administered together with peptide recognized in the context of HLA-DRB1 (41).

We first evaluated if vaccination with WT1 peptide was reported to induce expansion of WT1-specific T cells. By analyzing the results published in four trials (23 patients) where WT1-specific T cells were estimated by tetramer analysis or ELIspot assay without *ex vivo* expansion (6, 8, 11, 14), we were able to estimate that WT1 vaccination resulted in an overall median fold expansion in WT1-specific T cell frequencies of 2.4, as compared with baseline. The tetramer positive T cells increased from a median of 0.14% (range 0–0.98%) pre vaccination to 0.41% (range 0–6.6%) post-vaccination. Rezvani et al. (11) reported that the absolute number of CD3^+^CD8^+^ WT1 tumor T cells increased from a median value of 95 per mL pre vaccination, (range 20–423 cells/mL), to 398 per mL after vaccination (range 98–4570 cells/mL).

Keilholz et al. (7) reported a significant increase in the median frequency of WT1 tetramer positive T cells in the bone marrow from 0.18% (week 0) to 0.41%, at week 18 after vaccination (*P* = 0.04). In the peripheral blood, WT1 tetramer positive cells were present at 0.12% at baseline, increasing to 0.28% at week 10, and persisting at stable levels (0.25%) at week 18, although these values did not reach statistically significance. The authors reported that only patients with low blast count in the bone marrow at baseline (<40% blasts, *n* = 9) had a statistically significant expansion in the peripheral blood of WT1 tetramer positive T cells after vaccination, as compared with patients with a high blast count (>50% blasts, *n* = 9); the median frequencies at week 0, 10, and 18 were 0.11, 0.30, and 0.46% (*P* < 0.01) vs. 0.12, 0.27, and 0.23%, in the two groups, respectively. Interestingly, four patients in the low blast group had a functional WT1 T cell response [gamma-interferon (IFN)-gamma production] compared with only one patient in the high blast group. In all the evaluable patients from these studies, although WT1-specific T cells were present *in vivo* at low frequencies prior to vaccination, the functional response after WT1 peptide stimulation measured as IFN-gamma production was limited or absent, increasing only after vaccination.

In the study by Maslak et al. (13), a combination of WT1 peptides comprising of one short peptide with a mutated R126Y (heteroclitic) epitope (to elicit CD3^+^CD8^+^ T cells), two long peptides (to elicit CD3^+^CD4^+^ T cells), and one long peptide with the heteroclitic sequence (to elicit both CD3^+^CD4^+^ and CD3^+^CD8^+^ T cells) were tested. *Ex vivo* experiments with CD3^+^CD4^+^ T cells isolated from vaccinated patients showed that WT1-specific functional responses were stronger against the CD4 epitopes, although one patient showed a strong response toward the CD3^+^CD8^+^ heteroclitic-WT1_126–134_ peptide. Interestingly, long peptides elicited the strongest immunological responses *in vitro*, and an IFN-gamma ELIspot assay performed after two rounds of *in vitro* stimulation showed that both native and heteroclitic peptides could elicit strong functional responses against WT1. Although the HLA-DR heteroclitic peptide was more efficient than its native counterpart, both elicited responses against both the HLA-A02-01 and the HLA-DRB1 epitopes, indicating efficient processing and presentation of the HLA-A02-01 epitope embedded within the long peptide to CD8^+^ T cells.

Clinical responses and clinical benefit were observed in these studies, as reported in detail in Table 2, with some patients achieving and maintaining remission long-term (more than 8 years) (9). Of note, one patient had a complete response after the percentage of bone marrow blasts had reached 30% (7).

We also assessed whether correlation between WT1 responses and prognosis was reported in any of these studies. Some of the reviewed studies found a significant correlation between the detection of WT1-specific T cells and normalization/reduction of WT1 mRNA level [*P* < 0.01 (7, 11); *P* = 0.0397 (8)], whereas the loss of WT1-specific T cells was associated with reappearance of the WT1 transcript (11). A significant correlation was also reported between WT1 mRNA level and progression-free survival (*P* = 01), in one study (7).

Interestingly, in one study relapse was associated with the disappearance of T cell receptor clone restricted for Vbeta11 chain from the bone marrow (32), and a bias toward Vbeta11 usage of the WT1-specific T cells was further observed in four patients (33). In one patient, down-regulation of WT1 mRNA and loss of WT1 expression was observed at the moment of leukemia progression. However, additional immune-evasion mechanisms, such as WT1 mutation or loss of HLA expression on the surface of leukemic cells were not observed (34). Addressing other possible mechanisms resulting in loss of response to vaccination, Rezvani et al. (10) reported that repeated vaccinations eventually led to selective deletion of high avidity PR1- and WT1-specific CD3^+^CD8^+^ T cells and was not associated with significant reduction in WT1 expression.

Additional boosting failed to increase vaccine-induced WT1^+^CD8^+^T cell frequencies further and in all patients the response was lost before the sixth vaccine dose. Furthermore, the authors of another report suggested a negative impact of using the immunostimulatory toll like receptor 9 (TLR9) agonist oligodeoxynucleotide (CPG7909), and Montanide ISA51 (mISA51) as adjuvants for the vaccination (5).

Finally, in all the analyzed studies, vaccination with WT1 was found to be safe and well tolerated, with only 8% of patients (7 out of 88 total patients with any diagnosis) experiencing grade III-IV toxicity.

## Conclusion

Around 50% of patients undergoing allogeneic HSCT for MDS/AML experience long-term disease-free survival (2, 3), unfortunately, a significant proportion of patients will succumb to disease relapse (2). Alternative strategies are therefore urgently needed to improve outcomes, while also lowering treatment related mortalities and morbidities. The encouraging results to date from immunotherapeutic approaches, such as vaccination strategies, suggest that this option may offer a promising strategy to reduce the risk of disease relapse.

From the reports analyzed in our review, it is evident that vaccination with WT1 epitopes was safe, feasible, and potentially able to mediate sustained immune responses in patient with MDS/AML. Although these preliminary findings are encouraging, limitations of this review include the low number of patients in some of the analyzed clinical trials, and a heterogeneous group of patients with two different diseases diagnosis.

Although antigen-specific T cells for example against WT1 (35) and PR1 (36) are present in the blood of healthy donors and transferred to the patient after allogeneic HSCT or donor lymphocyte infusion, their persistence and expansion are transient, which may be explained by activation-induced apoptosis after exposure to high antigenic burden (37), or terminally differentiated effector memory phenotype (38). Therefore, vaccination approaches can potentially enhance anti-TAA immune responses. However, a comprehensive understanding of the mechanisms underlying a successful vaccine-induced immune response and of the factors predictive of response would allow the design of optimal immunotherapeutic strategies for the treatment of patients with MDS/AML.

Administration of large or repeated doses of foreign antigens in order to enhance effectiveness of the vaccine proved not beneficial in our experience (10), as it led to induction of immune tolerance, potentially via T cell deletion, anergy, or expansion of antigen-specific regulatory T cells (31).

An alternative approach to counteract immune-evasion mechanisms, such as down-regulation of TAA expression, would be to combine different epitopes of the antigen of interest. Two reports summarized here, including one from our own group, explored the feasibility of vaccinating patients with epitopes derived from two different TAAs, however larger or randomized clinical trials are needed to demonstrate the superiority of this approach (5, 10).

Two strategies to help circumvent the need for T-helper cells with resulting more sustained anti-cancer T cell immunity have been investigated with success in murine models (39, 40): (i) the adoption of synthetic long-sequence peptides, and (ii) the use of adjuvants to stimulate APCs.

Synthetic long-sequence peptides are preferentially processed by professional APCs in the lymph node draining area, circumventing some of the tolerance mechanisms. In the study of Maslak et al. (13), long peptides with capacity to elicit both a CD3^+^CD8^+^ and a CD3^+^CD4^+^ T cell response, resulted in stronger immunological responses *in vitro*, but whether this strategy would prove effective *in vivo* is yet to be established.

Although agonistic anti-CD40 antibodies induced maturation of APCs preventing tolerance induction and circumventing the need for CD4^+^ T cell help in the early phase of T cell response, it did not prevent the long-term induction of tolerance, likely because once the anti-CD40 antibody had been cleared, peptides were presented to CD8^+^ T cells by tolerogenic APCs (41). One possible strategy to sustain antigen exposure with APCs in the draining lymph node would be to combine the peptide with lipid tails (31), and this approach has been investigated with encouraging results using FDA approved biodegradable polylactic-co-glycolic acid microparticles, which shuttle antigens to the lymph nodes (42). To note, the replacement of mineral oils with novel delivery systems or the direct injection of peptides into lymph nodes (43) would also help in overcoming the long-term side effect of granuloma formation at the injection site observed with mISA51 (31).

Since persistence of antigen-specific T cells is required for successful immunotherapy, an optimal cytokine milieu (44, 45) or exogenous administration of cytokines (46) may result in preferential expansion of long-lived antigen-specific central memory T cells.

Priming donor T-lymphocytes *in vitro* to increase the frequency of tumor-specific precursors prior to adoptive transfer has been tested in murine models of leukemia (47), and has proven feasible in patients with multiple myeloma (48), and lymphoid neoplasms albeit without demonstration of clinical benefit in the latter population (49).

Additionally, in order to reduce the risk of inducing on-target off-tumor effects (50), the adoption of tumor-specific antigens, such as for example minor histocompatibility antigens selectively expressed by hematopoietic cells or exclusively expressed on hematopoietic progenitor cells, holds promise (51).

Finally, WT1 peptide vaccination strategies proved safe in this heterogeneous group of patient with MDS/AML. Although results from the reviewed studies suggest immunological and clinical benefit, with some patients experiencing long lived (more than 8 years) remissions of disease, more homogeneous and larger studies and randomized clinical trials are needed to quantify the contribution of WT1 peptide vaccines to clinical responses and disease-free survival.

## Author Contributions

All the authors contributed to conception, acquisition, and analysis of data, participated in the manuscript draft preparation, revision and approved, and revised the final version.

## Conflict of Interest Statement

The authors declare that the research was conducted in the absence of any commercial or financial relationships that could be construed as a potential conflict of interest.

## References

[1] Copelan EA Hematopoietic stem-cell transplantation. N Engl J Med (2006) 354:1813–26 10.1056/NEJMra052638 16641398

[2] Cornelissen JJ Gratwohl A Schlenk RF Sierra J Bornhauser M Juliusson G The European LeukemiaNet AML working party consensus statement on allogeneic HSCT for patients with AML in remission: an integrated-risk adapted approach. Nat Rev Clin Oncol (2012) 9:579–90. 10.1038/nrclinonc.2012.150 22949046

[3] Zittoun RA Mandelli F Willemze R de Witte T Labar B Resegotti L Autologous or allogeneic bone marrow transplantation compared with intensive chemotherapy in acute myelogenous leukemia. European organization for research and treatment of cancer (EORTC) and the gruppo Italiano malattie ematologiche maligne Dell’adulto (GIMEMA) leukemia cooperative groups. N Engl J Med (1995) 332:217–23. 7808487 10.1056/NEJM199501263320403

[4] Sekeres MA. The euphoria of hypomethylating agents in MDS and AML: is it justified? Best practice & research. Best Pract Res Clin Haematol (2013) 26:275–8. 10.1016/j.beha.2013.10.001 24309530

[5] Kuball J de Boer K Wagner E Wattad M Antunes E Weeratna RD Pitfalls of vaccinations with WT1-, Proteinase3- and MUC1-derived peptides in combination with MontanideISA51 and CpG7909. Cancer Immunol Immunother (2011) 60:161–71. 10.1007/s00262-010-0929-7 20963411 PMC3024516

[6] Mailander V Scheibenbogen C Thiel E Letsch A Blau IW Keilholz U Complete remission in a patient with recurrent acute myeloid leukemia induced by vaccination with WT1 peptide in the absence of hematological or renal toxicity. Leukemia (2004) 18:165–6 10.1038/sj.leu.2403186 14603333

[7] Keilholz U Letsch A Busse A Asemissen AM Bauer S Blau IW A clinical and immunologic phase 2 trial of Wilms tumor gene product 1 (WT1) peptide vaccination in patients with AML and MDS. Blood (2009) 113:6541–8. 10.1182/blood-2009-02-202598 19389880

[8] Oka Y Tsuboi A Taguchi T Osaki T Kyo T Nakajima H Induction of WT1 (Wilms’ tumor gene)-specific cytotoxic T lymphocytes by WT1 peptide vaccine and the resultant cancer regression. Proc Natl Acad Sci U S A (2004) 101:13885–90. 10.1073/pnas.0405884101 15365188 PMC518848

[9] Tsuboi A Oka Y Kyo T Katayama Y Elisseeva OA Kawakami M Long-term WT1 peptide vaccination for patients with acute myeloid leukemia with minimal residual disease. Leukemia (2012) 26:1410–3 10.1038/leu.2011.343 22157809

[10] Rezvani K Yong AS Mielke S Jafarpour B Savani BN Le RQ Repeated PR1 and WT1 peptide vaccination in Montanide-adjuvant fails to induce sustained high-avidity, epitope-specific CD8+ T cells in myeloid malignancies. Haematologica (2011) 96:432–40. 10.3324/haematol.2010.031674 21134985 PMC3046275

[11] Rezvani K Yong AS Mielke S Savani BN Musse L Superata J Leukemia-associated antigen-specific T-cell responses following combined PR1 and WT1 peptide vaccination in patients with myeloid malignancies. Blood (2008) 111:236–42. 10.1182/blood-2007-08-108241 17875804 PMC2200809

[12] Yasukawa M Fujiwara H Ochi T Suemori K Narumi H Azuma T Clinical efficacy of WT1 peptide vaccination in patients with acute myelogenous leukemia and myelodysplastic syndrome. Am J Hematol (2009) 84:314–5 10.1002/ajh.21387 19338044

[13] Maslak PG Dao T Krug LM Chanel S Korontsvit T Zakhaleva V Vaccination with synthetic analog peptides derived from WT1 oncoprotein induces T-cell responses in patients with complete remission from acute myeloid leukemia. Blood (2010) 116:171–9. 10.1182/blood-2009-10-250993 20400682 PMC2910606

[14] Hashii Y Sato-Miyashita E Matsumura R Kusuki S Yoshida H Ohta H WT1 peptide vaccination following allogeneic stem cell transplantation in pediatric leukemic patients with high risk for relapse: successful maintenance of durable remission. Leukemia (2012) 26:530–2 10.1038/leu.2011.226 21869838

[15] Schmitt M Schmitt A Rojewski MT Chen J Giannopoulos K Fei F RHAMM-R3 peptide vaccination in patients with acute myeloid leukemia, myelodysplastic syndrome, and multiple myeloma elicits immunologic and clinical responses. Blood (2008) 111:1357–65. 10.1182/blood-2007-07-099366 17978170

[16] Gessler M Poustka A Cavenee W Neve RL Orkin SH Bruns GA. Homozygous deletion in Wilms tumours of a zinc-finger gene identified by chromosome jumping. Nature (1990) 343:774–8. 10.1038/343774a0 2154702

[17] Sugiyama H. Wilms’ tumor gene WT1: its oncogenic function and clinical application. Int J Hematol (2001) 73:177–87. 10.1007/BF02981935 11372729

[18] Davies R Moore A Schedl A Bratt E Miyahawa K Ladomery M Multiple roles for the Wilms’ tumor suppressor, WT1. Cancer Res (1999) 59:1747s–50s. 10197591

[19] Moore AW McInnes L Kreidberg J Hastie ND Schedl A. YAC complementation shows a requirement for Wt1 in the development of epicardium, adrenal gland and throughout nephrogenesis. Development (1999) 126:1845–57. 10101119 10.1242/dev.126.9.1845

[20] Menssen HD Renkl HJ Rodeck U Maurer J Notter M Schwartz S Presence of Wilms’ tumor gene (wt1) transcripts and the WT1 nuclear protein in the majority of human acute leukemias. Leukemia (1995) 9:1060–7. 7596170

[21] Inoue K Sugiyama H Ogawa H Nakagawa M Yamagami T Miwa H WT1 as a new prognostic factor and a new marker for the detection of minimal residual disease in acute leukemia. Blood (1994) 84:3071–9. 7949179

[22] Tamaki H Ogawa H Ohyashiki K Ohyashiki JH Iwama H Inoue K The Wilms’ tumor gene WT1 is a good marker for diagnosis of disease progression of myelodysplastic syndromes. Leukemia (1999) 13:393–9. 10.1038/sj.leu.2401341 10086730

[23] Rauscher FJ III. The WT1 Wilms tumor gene product: a developmentally regulated transcription factor in the kidney that functions as a tumor suppressor. FASEB J (1993) 7:896–903. 8393820

[24] Haber DA Park S Maheswaran S Englert C Re GG Hazen-Martin DJ WT1-mediated growth suppression of Wilms tumor cells expressing a WT1 splicing variant. Science (1993) 262:2057–9. 10.1126/science.8266105 8266105

[25] Sugiyama H. Cancer immunotherapy targeting Wilms’ tumor gene WT1 product. Expert Rev Vaccines (2005) 4:503–12. 10.1586/14760584.4.4.503 16117707

[26] Algar EM Khromykh T Smith SI Blackburn DM Bryson GJ Smith PJ. A WT1 antisense oligonucleotide inhibits proliferation and induces apoptosis in myeloid leukaemia cell lines. Oncogene (1996) 12:1005–14. 8649791

[27] Nishida S Hosen N Shirakata T Kanato K Yanagihara M Nakatsuka S AML1-ETO rapidly induces acute myeloblastic leukemia in cooperation with the Wilms tumor gene, WT1. Blood (2006) 107:3303–12. 10.1182/blood-2005-04-1656 16380455

[28] Gaiger A Reese V Disis ML Cheever MA. Immunity to WT1 in the animal model and in patients with acute myeloid leukemia. Blood (2000) 96:1480–9. 10942395

[29] Gao L Bellantuono I Elsasser A Marley SB Gordon MY Goldman JM Selective elimination of leukemic CD34(+) progenitor cells by cytotoxic T lymphocytes specific for WT1. Blood (2000) 95:2198–203. 10733485

[30] Sugiyama H. WT1 (Wilms’ tumor gene 1): biology and cancer immunotherapy. Jpn J Clin Oncol (2010) 40:377–87. 10.1093/jjco/hyp194 20395243

[31] van Hall T van der Burg SH. Mechanisms of peptide vaccination in mouse models: tolerance, immunity, and hyperreactivity. Adv Immunol (2012) 114:51–76. 10.1016/B978-0-12-396548-6.00003-2 22449778

[32] Ochsenreither S Fusi A Busse A Bauer S Scheibenbogen C Stather D “Wilms Tumor Protein 1” (WT1) peptide vaccination-induced complete remission in a patient with acute myeloid leukemia is accompanied by the emergence of a predominant T-cell clone both in blood and bone marrow. J Immunother (2011) 34:85–91. 10.1097/CJI.0b013e3181f3cc5c 21150716

[33] Ochsenreither S Fusi A Geikowski A Stather D Busse A Stroux A Wilms’ tumor protein 1 (WT1) peptide vaccination in AML patients: predominant TCR CDR3beta sequence associated with remission in one patient is detectable in other vaccinated patients. Cancer Immunol Immunother (2012) 61:313–22. 10.1007/s00262-011-1099-y 21898091 PMC11029123

[34] Busse A Letsch A Scheibenbogen C Nonnenmacher A Ochsenreither S Thiel E Mutation or loss of Wilms’ tumor gene 1 (WT1) are not major reasons for immune escape in patients with AML receiving WT1 peptide vaccination. J Transl Med (2010) 8:5. 10.1186/1479-5876-8-5 20092642 PMC2844374

[35] Rezvani K Brenchley JM Price DA Kilical Y Gostick E Sewell AK T-cell responses directed against multiple HLA-A*0201-restricted epitopes derived from Wilms’ tumor 1 protein in patients with leukemia and healthy donors: identification, quantification, and characterization. Clin Cancer Res (2005) 11:8799–807. 10.1158/1078-0432.CCR-05-1314 16361568

[36] Rezvani K Price DA Brenchley JM Kilical Y Gostick E Sconocchia G Transfer of PR1-specific T-cell clones from donor to recipient by stem cell transplantation and association with GvL activity. Cytotherapy (2007) 9:245–51. 10.1080/14653240701218524 17464756

[37] Molldrem JJ Lee PP Kant S Wieder E Jiang W Lu S Chronic myelogenous leukemia shapes host immunity by selective deletion of high-avidity leukemia-specific T cells. J Clin Invest (2003) 111:639–47. 10.1172/JCI200316398 12618518 PMC151894

[38] Brenchley JM Karandikar NJ Betts MR Ambrozak DR Hill BJ Crotty LE Expression of CD57 defines replicative senescence and antigen-induced apoptotic death of CD8+ T cells. Blood (2003) 101:2711–20. 10.1182/blood-2002-07-2103 12433688

[39] Zwaveling S Ferreira Mota SC Nouta J Johnson M Lipford GB Offringa R Established human papillomavirus type 16-expressing tumors are effectively eradicated following vaccination with long peptides. J Immunol (2002) 169:350–8. 10.4049/jimmunol.169.1.350 12077264

[40] Cho HI Celis E. Optimized peptide vaccines eliciting extensive CD8 T-cell responses with therapeutic antitumor effects. Cancer Res (2009) 69:9012–9. 10.1158/0008-5472.CAN-09-2019 19903852 PMC2789207

[41] Bijker MS van den Eeden SJ Franken KL Melief CJ Offringa R van der Burg SH. CD8+ CTL priming by exact peptide epitopes in incomplete Freund’s adjuvant induces a vanishing CTL response, whereas long peptides induce sustained CTL reactivity. J Immunol (2007) 179:5033–40. 10.4049/jimmunol.179.8.5033 17911588

[42] Ali OA Doherty E Bell WJ Fradet T Hudak J Laliberte MT Biomaterial-based vaccine induces regression of established intracranial glioma in rats. Pharm Res (2011) 28:1074–80. 10.1007/s11095-010-0361-x 21225320

[43] Ribas A Weber JS Chmielowski B Comin-Anduix B Lu D Douek M Intra-lymph node prime-boost vaccination against Melan A and tyrosinase for the treatment of metastatic melanoma: results of a phase 1 clinical trial. Clin Cancer Res (2011) 17:2987–96. 10.1158/1078-0432.CCR-10-3272 21385924

[44] Kaneko S Mastaglio S Bondanza A Ponzoni M Sanvito F Aldrighetti L IL-7 and IL-15 allow the generation of suicide gene-modified alloreactive self-renewing central memory human T lymphocytes. Blood (2009) 113:1006–15. 10.1182/blood-2008-05-156059 18978209

[45] Rapoport AP Stadtmauer EA Aqui N Badros A Cotte J Chrisley L Restoration of immunity in lymphopenic individuals with cancer by vaccination and adoptive T-cell transfer. Nat Med (2005) 11:1230–7. 10.1038/nm1310 16227990

[46] Ansell SM Witzig TE Kurtin PJ Sloan JA Jelinek DF Howell KG Phase 1 study of interleukin-12 in combination with rituximab in patients with B-cell non-Hodgkin lymphoma. Blood (2002) 99:67–74. 10.1182/blood.V99.1.67 11756154

[47] Kohrt HE Muller A Baker J Goldstein MJ Newell E Dutt S Donor immunization with WT1 peptide augments antileukemic activity after MHC-matched bone marrow transplantation. Blood (2011) 118:5319–29. 10.1182/blood-2011-05-356238 21868578 PMC3217412

[48] Neelapu SS Munshi NC Jagannath S Watson TM Pennington R Reynolds C Tumor antigen immunization of sibling stem cell transplant donors in multiple myeloma. Bone Marrow Transplant (2005) 36:315–23. 10.1038/sj.bmt.1705057 15968284

[49] Levy R Ganjoo KN Leonard JP Vose JM Flinn IW Ambinder RF Active idiotypic vaccination versus control immunotherapy for follicular lymphoma. J Clin Oncol (2014) 32:1797–803. 10.1200/JCO.2012.43.9273 24799467 PMC4039868

[50] Falkenburg WJ Melenhorst JJ van de Meent M Kester MG Hombrink P Heemskerk MH Allogeneic HLA-A*02-restricted WT1-specific T cells from mismatched donors are highly reactive but show off-target promiscuity. J Immunol (2011) 187:2824–33. 10.4049/jimmunol.1100852 21821799

[51] Marijt WA Heemskerk MH Kloosterboer FM Goulmy E Kester MG van der Hoorn MA Hematopoiesis-restricted minor histocompatibility antigens HA-1- or HA-2-specific T cells can induce complete remissions of relapsed leukemia. Proc Natl Acad Sci U S A (2003) 100:2742–7. 10.1073/pnas.0530192100 12601144 PMC151411

